# The First Case of *Streptococcus sinensis* Endocarditis in Italy: Case Presentation and Systematic Literature Review

**DOI:** 10.3390/medicina60121991

**Published:** 2024-12-02

**Authors:** Maria Mazzitelli, Maria Teresa Sartori, Vincenzo Scaglione, Fabrizio Lucente, Gino Gerosa, Andrea Leonardi, Ignazio Castagliuolo, Valeria Pergola, Paolo Simioni, Annamaria Cattelan

**Affiliations:** 1 Infectious and Tropical Diseases Unit, Padua University Hospital, 35128 Padua, Italyannamaria.cattelan@aopd.veneto.it (A.C.); 2 Department of Medicine—DIMED, Padua University Hospital, 35128 Padua, Italy; mariateresa.sartori@aopd.veneto.it (M.T.S.); fabrizio.lucente@aopd.veneto.it (F.L.);; 3 Cardiac Surgery Unit, Department of Cardiac, Thoracic, Vascular Sciences and Public Health Padua University Hospital, 35128 Padua, Italy; 4 Ophthalmology Unit, Department of Neuroscience, Padua University Hospital, 35128 Padua, Italy; 5 Microbiology and Virology Unit, Padua University Hospital, 35128 Padua, Italy; 6 Cardiology Unit, Department of Cardiac Thoracic Vascular Sciences and Public Health, Padua University Hospital, 35128 Padua, Italy; valeria.pergola@aopd.veneto.it

**Keywords:** *Streptococcus sinensis*, endocarditis, emerging pathogen, review

## Abstract

Almost 25 years have now passed since the first identification of *Streptococcus sinensis* (*S. sinensis*). It can cause infections both in immunocompetent and immunocompromised hosts. However, it has been rarely described as an aetiology of infectious endocarditis. We herein report the case of a 75-year-old Italian gentleman who was admitted for shortness of breath, asthenia, weight loss, and an episode of loss of consciousness and who was subsequently diagnosed with *S. sinensis* endocarditis (the first reported in Italy). He was, therefore, treated with ceftriaxone for six weeks and underwent cardiac surgery. We performed a literature review on *S. sinensis* endocarditis cases and found 12 other reported cases. Demographics, clinical presentation, prognostic factors, treatment, and outcomes were summarized. Despite anecdotic cases being reported, *S. sinensis* endocarditis can occur and should be promptly and properly identified using accurate diagnostic methods. Continued research into its epidemiology, pathogenesis, antimicrobial resistance, and host interactions is essential for enhancing our knowledge and improving clinical management strategies.

## 1. Introduction

*Streptococcus sinensis* (*S. sinensis*), a member of the *Streptococcus mitis* group, has emerged as an intriguing bacterium in the realm of clinical microbiology since its initial characterization in 2001 by Collins et al. [1]. This novel species has attracted significant attention due to its unique microbiological features, clinical significance, and prevalence in human microbial communities. *S. sinensis* is a Gram-positive, non-motile, catalase-negative, facultatively anaerobic bacterium belonging to the *phylum Firmicutes*, characterized by partial hemolysis, indicative of its ability to degrade hemoglobin [2,3]. *S. sinensis* diagnosis has been implemented through 16S rRNA or MALDI-TOF Mass Spectrometry [4,5]. Furthermore, whole-genome sequencing has provided insights into the genomic architecture of *S. sinensis*, revealing its genetic composition, metabolic pathways, and potential virulence factor [6].

While closely related to other *viridans* Streptococci, *S. sinensis* exhibits several distinctive features that set it apart within the *Streptococcus* genus, including its biochemical profile, antigenic properties, and susceptibility to antimicrobial agents [6].

Phenotypically, *S. sinensis* demonstrates unique fermentation patterns for carbohydrates and distinctive enzyme activities, contributing to its differentiation from phylogenetically related species. Additionally, *S. sinensis* possesses surface antigens and adhesins that mediate its interaction with host tissues and immune evasion strategies. Studies have identified surface proteins involved in adhesion to epithelial cells and extracellular matrix components, potentially influencing its colonization and pathogenicity. Antimicrobial susceptibility testing has revealed varying resistance profiles among clinical isolates of *S. sinensis*, highlighting the importance of accurate susceptibility testing to guide antimicrobial therapy. While generally susceptible to beta-lactam antibiotics, resistance to other antibiotic classes, such as macrolides and fluoroquinolones, has been reported, necessitating vigilant surveillance of antimicrobial resistance patterns.

*S. sinensis* is primarily found as a commensal organism in the oropharynx and upper respiratory tract of humans. Epidemiological studies have documented its prevalence in healthy individuals as well as in patients with underlying medical conditions. While the exact prevalence varies across populations and geographical regions, *S. sinensis* is commonly isolated from respiratory specimens, saliva, dental plaques, and nasopharyngeal swabs. Moreover, *S. sinensis* has been implicated in various clinical infections, including endocarditis, bacteremia, pneumonia, and deep-seated abscesses, underscoring its pathogenic potential. The prevalence of *S. sinensis* in clinical settings highlights the need for accurate species identification and characterization to facilitate targeted therapeutic interventions and infection control measures, and to date, very few cases have been reported [7,8,9,10,11,12,13,14,15,16].

We herein describe the first case of endocarditis, defined as per Duke criteria [17], by *S. sinensis* reported in Italy. Through a systematic literature review, we conduct an analysis of the clinical characteristics and outcomes associated with *S. sinensis* endocarditis.

## 2. Case Presentation

A 75-year-old Italian gentleman was hospitalized because of a 4-week history of exertional dyspnoea, profuse asthenia, progressive loss of appetite and weight loss of approximately 10 Kg, and an episode of loss of consciousness. He had no history of travels or anything relevant to disclose in his recent medical history. His past medical history revealed acute promyelocytic leukemia successfully treated in 2004, aortoiliac bypass surgery for an infrarenal abdominal aortic aneurysm in 2007, and radiotherapy in 2014 for prostate adenocarcinoma. He was also suffering from dyslipidemia on treatment with rosuvastatin 10 mg/day, depression on treatment with sertraline 50 mg/day, diabetes on treatment with empagliflozin 10 mg/day, and hyperthyroidism on treatment with methimazole 5 mg/day. At admission, his temperature was 38.5 °C, his pulse was 75 beats per minute with extrasystoles, his blood pressure was 130/60 mmHg, his respiratory rate was 15 breaths per minute, and his oxygen saturation was 99% in room air. The blood tests showed a normal white blood count with anemia (hemoglobin = 85 g/L), for which the patient received a blood transfusion. The plasma C-reactive (CPR) protein level was 39 mg/L (normal values < 0.5 mg/L), and the procalcitonin level was within the normal range. There was a slight impairment in kidney function, with an eGFR of 48 mL/min and a creatinine level of 110 mmol/L. The liver function tests were normal. In addition, two sets of blood cultures were performed during the febrile episode.

The physical examination revealed a systolic murmur 3/4 Levine prevalent in the mesocardium, raising the suspicion of infectious endocarditis, despite that the patient had no history of recent dental procedures, rheumatic heart disease, or other conditions that could predispose him to the ongoing condition. No cutaneous signs of infectious endocarditis, such as Janeway lesions, Osler nodes, or splinter hemorrhages beneath the fingernails, were detected, and the rest of the clinical assessment was unremarkable. Chest X-ray was negative, as was a pulmonary angio-CT scan performed for a mild elevation of d-dimer (3699 mcg/L, normal values <700) to exclude pulmonary embolism.

**Figure 1:**
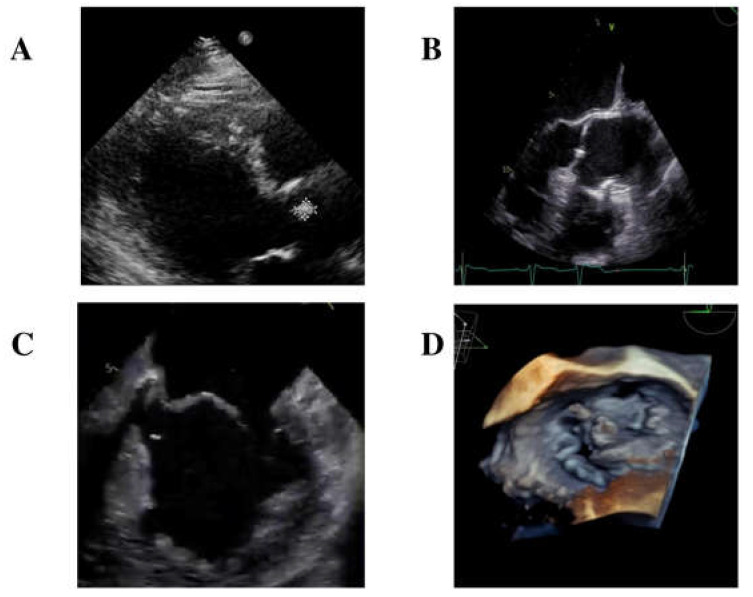
Transthoracic echocardiogram showing a mobile isoechoic formation at the right cuspid of the aortic valve (**A**). Transoesophageal echocardiogram image depicting a mobile isoechoic formation measuring 0.7 mm × 0.7 mm on the right cusp of the aortic valve (**B**); a mobile isoechoic formation on the posterior mitral leaflet with fragmentary flail of P2 due to rupture of the chordae tendineae (**C**); three-dimensional imaging of the mitral valve with the mass seen on P2 scallop (**D**).

A transthoracic echocardiogram identified a small isoechoic mass (0.7 mm × 10 mm) on the right cusp of the aortic valve (Figure 1A) and diagnosed mitral valve dysfunction with severe regurgitation, requiring the initiation of diuretic therapy.

Furthermore, an empiric antibiotic treatment with intravenous ceftriaxone 2 gr/day was started. In the meanwhile, blood cultures came back positive for Gram-positive rods, and MALDI-TOF identified *S. sinensis*. Further diagnostic evaluations included a transesophageal echocardiogram (TEE), which confirmed the presence of the isoechoic mass measuring 0.7 mm × 0.7 mm on the right cusp of the aortic valve (Figure 1B). Additionally, TEE identified another long (length = 12.5 mm) mobile iso-echogenic mass on the medial scallop of the posterior mitral leaflet (P2), with a flail of P2 due to rupture of the chordae tendineae (Figure 1C,D).

**Figure 2:**
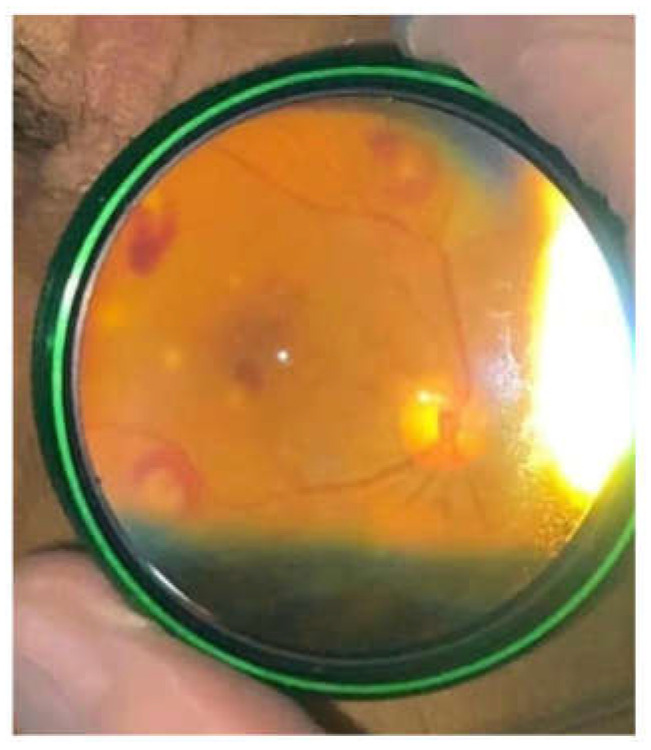
Left eye fundoscopy at the bedside of the patient revealed multiple Roth dots (superficial retinal hemorrhages ovally-shaped, with pale centre) and white retinal infiltrates at the posterior pole.

A bedside ophthalmic examination conducted during the hospital stay revealed multiple Roth dots (oval and superficial retinal hemorrhages, with pale center) in the left eye and white retinal infiltrates at the posterior pole, consistent with infectious embolization (Figure 2). The case was hence defined as certain *S. sinensis* endocarditis, according to Duke criteria-ISCVID 2023, with the presence of both major and minor criteria.

CT scans of the brain and abdomen were both negative for embolization. According to the antimicrobial susceptibility testing results, antibiotic therapy with ceftriaxone was continued for a total of 6 weeks. Follow-up blood cultures performed 3 days after the start of the antibiotic confirmed the clearance of the bacteremia. The patient’s condition gradually improved as well as the inflammatory biomarkers. He then underwent a cardiac surgery procedure, which included a successful mitral prosthetic valve replacement. The intervention was complicated by mild bleeding, which required mediastinal revision surgery, but the post-operative course was normal and with a favourable evolution. The patient was discharged home eleven weeks after admission, and follow-up cardiac US performed one month and three months after discharge was negative. To date, the patient is in good clinical condition.

## 3. Systematic Literature Review: Methodology and Results

The Preferred Reporting Items for Systematic Review and Meta-Analysis (PRISMA) guidelines were followed to perform the review. The Embase/MEDLINE database was screened backward from 15 July 2024. The search was conducted by using the words “Endocarditis” or “heart infections” AND “*Streptococcus sinensis*” OR “*S. sinensis*”. Two reviewers (MM and VS) independently screened the titles and abstracts to deem eligibility for full-text assessment. No language or geographical restrictions were applied. We included all papers that included cases published as full articles/case reports/systematic reviews about *S. sinensis* endocarditis. From each paper, we extracted, whenever available, information about demographics (gender, age, country), comorbidities, risk factors, symptom onset, diagnosis, treatment, and clinical outcome.

We described the first case of infectious endocarditis by *S. sinensis* ever reported in Italy. The other 12 cases reported in the literature are described in Table 1. Cases reported, including ours, are mostly male (7/13, 53%) and with an age range from 19 to 75 years, with our case being the oldest ever reported.

*S. sinensis* was isolated for the first time in 2002 in a 42-year-old Chinese woman diagnosed with endocarditis [7]. It is worth mentioning that most cases have primarily been observed in Southeast Asia, particularly in the Hong Kong region [7,13]. However, there have been more recent reports of cases in Europe [8,9,10,11,12]. Out of the 12 clinical cases documented in the literature, 8 people were from Asia, 1 was from Europe, and in 3 cases, the origin was not available. Additionally, while in most cases, a travel history was reported, in our case, there was no mention of travel. This underscores the significance of determining the travel history of those with *S. sinensis* infection to identify geographical sources for this pathogen, highlighting its potential as a new emerging infectious agent, but at the same time, it is worth considering the oral source as the main driver. The median time interval between symptoms onset and infectious endocarditis diagnosis in eight out of nine individuals was 10 weeks (range: 1–36 weeks), categorizing them as late-diagnosed infectious endocarditis cases (diagnosis occurring more than 1 month after initial symptoms), accounting for approximately 45% of all bacterial IE cases [18]. As already stated by Zhang et al. in their review [15], previous *S. sinensis* clinical cases lack in-depth characterizations, and it is not possible to speculate much about prognostic factors and their correlation with treatment outcomes. Most cases described had predisposing risk factors for developing infectious endocarditis, such as rheumatic heart disease, dental procedures, or valve alterations (either congenital or degenerative). The median antibiotic treatment length was 6 weeks and was mainly based on combination therapy of penicillin or ceftriaxone with gentamycin, while monotherapy with ceftriaxone was used only in two cases. Most patients underwent cardiac surgery either due to significant valve dysfunction or the size of vegetations, except for one patient who was planned for intervention but could not undergo it due to the severity of clinical presentation and personal beliefs, and three for whom this information is not available. Embolization phenomena were reported in 7 cases (53.8%), primarily affecting the central nervous system and eye.

Unfortunately, one case resulted in a fatality due to central nervous system embolization complications. The overall mortality rate was 7.7% (1/13), which is relatively lower compared to other pathogens like *Enterococci* (15–25%), *Staphylococcus aureus* (25–47%), *Pseudomonas aeruginosa*, and fungal species (50%) [19].

## 4. Discussion

The clinical significance of *S. sinensis* infection relies on its potential to cause severe infections, particularly in immunocompromised individuals or those with predisposing risk factors.

To date, the most common clinical manifestations of *S. sinensis* infections include bloodstream infections and endocarditis [13]. Both require prompt diagnosis and aggressive management, including surgery, to avoid further dissemination of the infection, which often causes central nervous system dissemination. In addition, timely identification of the causative organism and assessment of antimicrobial susceptibility are crucial for enhancing patient outcomes.

*S. sinensis*, along with *Streptococcus cristatus,* is classified into the “cristatus clade” of the *S. mitis* group of the streptococci genus, according to the new phylogenetic classification [5]. Despite its microbiological features, the treatment of *S. sinensis* endocarditis does not differ much from cases caused by other strains from the same group. The oral cavity seems to be the natural reservoir of this bacterium, similar to other *viridans* Streptococci. It was detected in 22% of saliva samples from 100 healthy volunteers, and it was probably the apparent source of the infection in patients with infective endocarditis [19]. Indeed, in our cases, the history of travel, which has been mentioned in other cases as a major risk factor, is lacking, making it more probable that the etiology came from the oral cavity. However, global concerns are emerging since most cases were detected in Asia, but the number of cases in Europe is rising. Currently, *S. sinensis* is very sensitive to penicillin, ceftriaxone, cefepime, clindamycin, erythromycin, ofloxacin, tetracycline, and vancomycin [7,20,21]. However, the ability of *S. sinensis* to form biofilms on host surfaces further exacerbates its pathogenic potential, rendering it more resistant to host immune defenses and antimicrobial agents, particularly in endocarditis cases [22]. In fact, most of the reported cases underwent surgery due to severe native valve disruption. All cases reported involved native valves, but the latest recently published for which the bioprosthetic aortic valve was involved, posing further challenges in clinical management [16].

*S. sinensis* has been implicated in polymicrobial infections, where it coexists with other pathogenic or commensal microorganisms. Coexisting pathogens complicate further clinical management and the selection of appropriate antimicrobial therapy.

## 5. Conclusions

In conclusion, *S. sinensis* represents a fascinating microorganism with distinctive microbiological features, clinical significance, and prevalence in human microbial communities. Continued research into its epidemiology, pathogenesis, antimicrobial resistance, and host interactions is essential for enhancing our understanding of this emerging pathogen and improving clinical management strategies. By elucidating the complex interplay between *S. sinensis* and the host environment, we can develop more effective preventive measures and therapeutic interventions to mitigate the impact of *S. sinensis*-associated infections on human health.

## Figures and Tables

**Table 1:** Endocarditis cases by *S. sinensis*.

Authors	Year (Publication)	n. Cases	Gender	Age	Country	Origin of the Case	Interval Between Symptom Onset and Diagnosis	Valve Involvement	Antibiotic Treatment	Length Antibiotic Treatment	Embolization	Cardiac Surgery	Congenital Heart Disease/Underlying Conditions	Outcome
Woo PC et al. [7]	2002	1	F	42	China	Chinese	1 week	No	Ampicillin, gentamycin	6 weeks	Janeway lesion on the left palm	NA	Chronic rheumatic heart disease	Survived
Woo PC et al. [6,8]	2004, 2006	3	NA	NA	China	NA	NA	Yes (for 1 not specified)	Penicillin G/Ampicillin/gentamycin	NA	1 embolization; for the other 2 NA	NA	Chronic rheumatic heart disease in all cases, 1 dental procedure	NA
Uckay et al. [9]	2007	1	M	57	Switzerland	Italian	4 weeks	Yes	Penicillin, gentamycin	6 weeks	Embolus near the right macula	Yes	Chronic rheumatic heart disease, Dental procedures without prophylaxis	Survival
Faibis et al. [10]	2008	1	M	55	France	NA	12 weeks	Yes	Amoxicillin/clavulanate, gentamycin	8 weeks	No	Yes	Mitral valvular dystrophy	Survival
Seta et al. [11]	2015	1	F	20	France	Vietnam; recently arrived in France from Asia	Not known	No	Amoxicillin/clavulanate, gentamycin	4 weeks	No	Yes	No	Survival
San Francisco et al. [12]	2019	1	M	63	Britain	Extensive history of travel to Asia	36 weeks	No	Amoxicillin/clavulanate, gentamycin	6 weeks	Roth spot	Yes	No	Survival
Goret et al. [13]	2019	1	M	37	France	NA	12 weeks	Yes	Ceftriaxone	4 weeks	No	Yes	Six teeth requiring extraction	NA
Van Ommen et al. [14]	2020	1	M	58	Netherlands	Asian	12 weeks	Yes	Amoxicillin/clavulanate, gentamycin	1 week	Cerebral abscess and infarction	No	NA	Death
Zhang et al. [15]	2022	1	M	19	Mainland China	NA	8 weeks	Yes	Penicillin, gentamycin	6 weeks	No	Yes	Aortic valve bicuspid malformation	Survival
Pan et al. [16]	2024	1	F	40	Asia	Chinese	45 days	Yes	Penicillin, ceftriaxone	6 weeks	Cutaneous lesions	Yes	Aortic valve replacement/dental care without prophylaxis	Survival
This case	2024	1	M	75	Italy	Italian; no travel history	4 weeks	Yes	Ceftriaxone	6 weeks	Eye	Yes	Dental care	Survival

M = male, F = female, NA = not available.

## Data Availability

All data used to generate this manuscript are herein reported.

## References

[1] Facklam R. (2002). What happened to the streptococci: Overview of taxonomic and nomenclature changes. Clin. Microbiol. Rev..

[2] Doern C.D., Burnham C.A. (2010). It’s not easy being green: The viridans group streptococci, with a focus on pediatric clinical manifestations. J. Clin. Microbiol..

[3] Zheng W., Tan T.K., Paterson I.C., Mutha N.V., Siow C.C., Tan S.Y., Old L.A., Jakubovics N.S., Choo S.W. (2016). StreptoBase: An Oral Streptococcus mitis Group Genomic Resource and Analysis Platform. PLoS ONE.

[4] Angeletti S., Dicuonzo G., Avola A., Crea F., Dedej E., Vailati F., Farina C., De Florio L. (2015). Viridans Group Streptococci clinical isolates: MALDI-TOF mass spectrometry versus gene sequence-based identification. PLoS ONE.

[5] Jensen A., Scholz C.F.P., Kilian M. (2016). Re-evaluation of the taxonomy of the Mitis group of the genus Streptococcus based on whole genome phylogenetic analyses, and proposed reclassification of Streptococcus dentisani as Streptococcus oralis subsp. dentisani comb. nov., Streptococcus tigurinus as Streptococcus oralis subsp. tigurinus comb. nov., and Streptococcus oligofermentans as a later synonym of Streptococcus cristatus. Int. J. Syst. Evol. Microbiol..

[6] Woo P.C., Teng J.L., Lau S.K., Yuen K.Y. (2006). Clinical, phenotypic, and genotypic evidence for Streptococcus sinensis as the common ancestor of anginosus and mitis groups of streptococci. Med. Hypotheses.

[7] Woo P.C., Tam D.M., Leung K.W., Lau S.K., Teng J.L., Wong M.K., Yuen K.Y. (2002). Streptococcus sinensis sp. nov., a novel species isolated from a patient with infective endocarditis. J. Clin. Microbiol..

[8] Woo P.C., Teng J.L.L., Leung K.W., Lau S.K.P., Tse H., Wong B.H.L., Yuen K.Y. (2024). Streptococcus sinensis may react with Lancefield group F antiserum. J. Clin. Med. Microbiol..

[9] Uckay I., Rohner P., Bolivar I., Ninet B., Djordjevic M., Nobre V., Garzoni C., Schrenzel J. (2007). Streptococcus sinensis endocarditis outside Hong Kong. Emerg. Infect. Dis..

[10] Faibis F., Mihaila L., Perna S., Lefort J.F., Demachy M.C., Le Fleche-Mateos A., Bouvet A. (2008). Streptococcus sinensis: An emerging agent of infective endocarditis. J. Med. Microbiol..

[11] Seta V., Teicher E., Fortineau N., Ladouceur M., Lambotte O. (2015). Infective endocarditis caused by Streptococcus sinensis. Med. Mal. Infect..

[12] San Francisco A., Tomlinson J.S., Walters S., Curtis S., James R. (2019). Lesson of the month 2, When steroids stop working—Infective endocarditis, the great mimicker. Clin. Med..

[13] Goret J., Baudinet T., Camou F., Issa N., Gaillard P., Wirth G., Greib C., Barandon L., Megraud F., Bebear C. (2019). Identification of Streptococcus sinensis from a patient with endocarditis using MALDI-TOF mass spectrometry, 16S rDNA- and sodA-based phylogeny. J. Microbiol. Immunol. Infect..

[14] van Ommen A., Slavenburg S., Diepersloot R., de Vries Feyens C.A. (2020). Fatal outcome of first case of Streptococcus sinensis in infective endocarditis in the Netherlands: A case report. Eur. Heart J. Case Rep..

[15] Zhang Y., Wang J., Zhan Y., Tang R., Wang H., Qin T., Lu Z. (2022). Case report: Infective endocarditis caused by Streptococcus sinensis: The first case in mainland China and literature review. Front. Cardiovasc. Med..

[16] Pan Y., Qian J., Wang G., Zhao H. (2024). Infective Endocarditis Caused by Streptococcus sinensis in a patient with Bioprosthetic Aortica Valve: A case report and literature review. Infect. Drug Res..

[17] Fowler V.G., Durack D.T., Selton-Suty C., Athan E., Bayer A.S., Chamis A.L., Dahl A., Dibernardo L., Durante-Mangoni E., Duval X. (2023). The 2023 Duke-ISCVID Criteria for Infective Endocarditis: Updating the Modified Duke Criteria. Clin. Infect. Dis..

[18] Bennani G., Zahri S., Habbal R. (2024). Time interval between first symptoms and diagnosis of infective endocarditis: Characteristics, microorganisms and impact on prognosis. Arch. Cardiovasc. Dis..

[19] Mylonakis E., Calderwood S.B. (2001). Infective endocarditis in adults. N. Engl. J. Med..

[20] Woo P.C., Teng J.L., Tsang S.N., Tse C.W., Lau S.K., Yuen K.Y. (2008). The oral cavity as a natural reservoir for Streptococcus sinensis. Clin. Microbiol. Infect..

[21] Singh N., Poggensee L., Huang Y., Evans C.T., Suda K.J., Bulman Z.P. (2022). Antibiotic susceptibility patterns of viridans group streptococci isolates in the United States from 2010 to 2020. JAC Antimicrob. Resist..

[22] Yadav P., Verma S., Bauer R., Kumari M., Dua M., Johri A.K., Yadav V., Spellerberg B. (2020). Deciphering Streptococcal Biofilms. Microorganisms.

